# Medical Astro-Microbiology: Current Role and Future Challenges

**DOI:** 10.1007/s41745-023-00360-1

**Published:** 2023-04-08

**Authors:** Francesca McDonagh, Martin Cormican, Dearbháile Morris, Liam Burke, Nitin Kumar Singh, Kasthuri Venkateswaran, Georgios Miliotis

**Affiliations:** 1 Antimicrobial Resistance and Microbial Ecology Group, School of Medicine, University of Galway, Galway, Ireland; 2 Department of Medical Microbiology, Galway University Hospitals, Galway, Ireland; 3 Biotechnology and Planetary Protection Group, NASA Jet Propulsion Laboratory, California Institute of Technology, Pasadena, CA USA

## Abstract

The second and third decades of the twenty-first century are marked by a flourishing of space technology which may soon realise human aspirations of a permanent multiplanetary presence. The prevention, control and management of infection with microbial pathogens is likely to play a key role in how successful human space aspirations will become. This review considers the emerging field of medical astro-microbiology. It examines the current evidence regarding the risk of infection during spaceflight via host susceptibility, alterations to the host’s microbiome as well as exposure to other crew members and spacecraft’s microbiomes. It also considers the relevance of the hygiene hypothesis in this regard. It then reviews the current evidence related to infection risk associated with microbial adaptability in spaceflight conditions. There is a particular focus on the International Space Station (ISS), as one of the only two  crewed objects in low Earth orbit. It discusses the effects of spaceflight related stressors on viruses and the infection risks associated with latent viral reactivation and increased viral shedding during spaceflight. It then examines the effects of the same stressors on bacteria, particularly in relation to changes in virulence and drug resistance. It also considers our current understanding of fungal adaptability in spaceflight. The global public health and environmental risks associated with a possible re-introduction to Earth of invasive species are also briefly discussed. Finally, this review examines the largely unknown microbiology and infection implications of celestial body habitation with an emphasis placed on Mars. Overall, this review summarises much of our current understanding of medical astro-microbiology and identifies significant knowledge gaps.

**Graphical Abstract:**

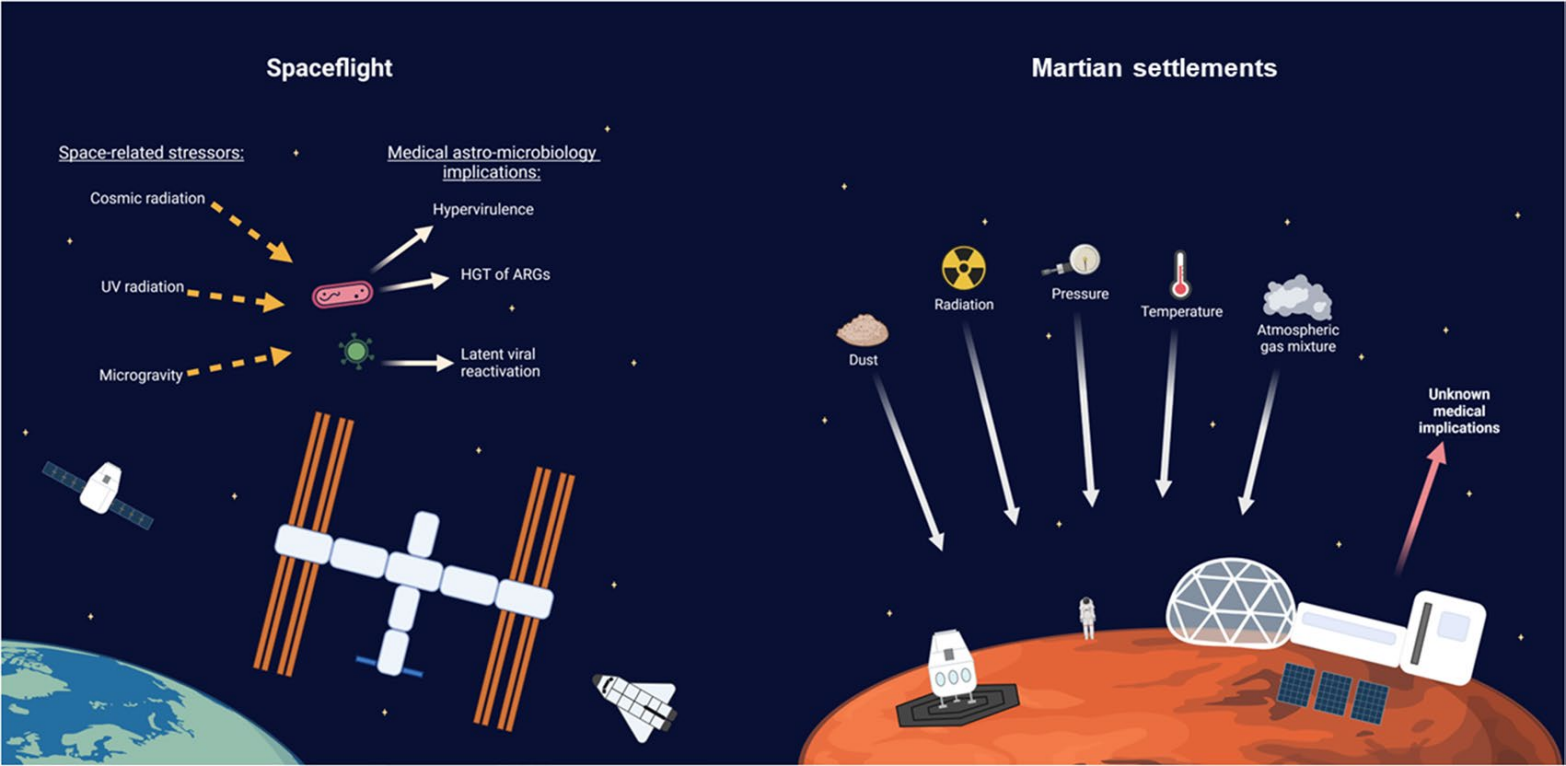

## Introduction

The emergence and dissemination of infectious disease has been a regular occurrence throughout human history and has led to pivotal societal, economic, and technologic changes. In premodern societies many infectious diseases such as cholera, polio, the plague, smallpox and typhus were endemic and/or caused periodic major epidemics resulting in extensive death, disability and societal disruption.^1^ Spread of infection was facilitated by conflict, colonisation, war, poverty, poor hygiene, inadequate sanitation, and absence of current technologies for detection, prevention and management of infection including modern-day medical advancements such as antibiotics and vaccines.^2^ Despite advances in the understanding and control of infectious disease, human society remains vulnerable to the emergence and dissemination of infection. New emerging infectious diseases (EIDs)^MN1^

^**MN1**^EIDs include newly identified infectious diseases, or known infectious diseases presenting an increase in population spread or geographic distribution.

have become increasingly recognised in recent decades.^3^ The rise of EIDs is likely related to population expansion, changes in agricultural practices to more intensive food production, climate change, urbanisation, an unprecedented level of global connectedness and complex/concentrated healthcare systems. These factors are also enabling infectious disease outbreaks to rapidly disseminate within the human population, allowing minimal time for effective containment of emerging infections.^4,5^ This continuing threat is illustrated by the dissemination of Severe Acute Respiratory Syndrome Coronavirus-2 (SARS-CoV-2), Mpox, Human Immunodeficiency Virus (HIV) and Carbapenemase-Resistant Enterobacterales (CRE) amongst others.^6–9^ Globalisation has also been associated with environmental risks related to invasive species (i.e., organisms not native in a particular area).^10^ The environmental and biodiversity impact of invasive macro^MN2^

^**MN2**^Macrofauna relates to organisms of a size of a few milimeters (mm) to several centimeters (cm) within an ecosystem. Megafauna relates to larger organisms.

and mega fauna^MN2^ and flora is readily apparent.^11,12^ The phenomenon of microbial invasive species is much less apparent and less well studied, but by extrapolation from the dissemination of antimicrobial resistant organisms (AROs) it is likely that globalisation also contributes to a rapid change and homogenisation in the global microbiome.^13^ In this context, careful consideration must be given to potential risks of emergence and spread of invasive microbial species associated with space travel and multi-planet settlement in the coming decades.

Space travel has so far been reserved for relatively few; predominantly specially selected and trained astronauts. The moderating agencies [e.g., National Aeronautics and Space Administration (NASA)] apply a series of measures to manage risk of infection such as those described in NASA’s Health Stabilisation Programme (HSP).^14^ In the coming decades, a shift towards space travel and multi-planetary space settlement is expected, with permanent human presence planned on the Moon and Mars within the next two decades.^15^ By 2050, SpaceX is estimating that thousands of people could be living and working on Mars. Schedules for smaller research-based outposts of 1000–2500 inhabitants are also anticipated for the Moon within the same timeframe. Regardless of the timeline feasibility of these projections, an extended permanent human space presence is anticipated within the next few decades.^16^ The challenges from a microbiological and infectious disease perspective are multifaceted. They firstly entail the protection of the space traveller from infection during spaceflight. Infection may originate from their own microbiome (e.g., reactivation of latent microorganisms),^17^ the microbiome of other space travellers or the environment.^18^ At the present time the environment is primarily the interior of constructed spacecrafts of terrestrial origin. Exposure in that context is expected to be to terrestrial organisms or to organisms of terrestrial origin that have been altered by selective forces encountered during spaceflight.^19^ In the future, when plans to populate the surface of other celestial bodies proceed there is a potential risk of exposure to extant extra-terrestrial microorganisms, if such life exists.^20^ Furthermore, such an encounter would have uncharted interactions with terrestrial microbial life forms and depending on genomic compatibility there might be a potential for terrestrial microorganisms to incorporate genetic elements of such extant organisms.

The larger overall challenge is the protection of global public health and the global environment from risks associated with return of vehicle, materials, and travellers to Earth. The more immediate risk could relate to the potential to introduce pathogenic and drug resistant invasive microorganisms that have adapted within a spacecraft environment and are capable of dissemination on Earth. The more remote possibility is the accidental introduction of extra-terrestrial microorganisms into Earth systems. The likelihood of introduction and escape of microorganisms that are well adapted to Earth environments and are harmful to human, animal or plant health (pathogenic) or to the environment (invasive species)^MN3^

^**MN3**^An organism that is not indigenous to the ecosystem of consideration.

is deemed to be low.^21^ On the other hand, the consequences of such introduction would be severe for public and environmental health as well as for the reputation and sustainability of space exploration. Furthermore, a permanent human presence on other celestial bodies (e.g., Mars) would certainly introduce terrestrial microorganisms to those settings.^22,23^ In the event of human travel, the introduction of terrestrial microorganisms is unavoidable as humans travel with their microbiome.^24^ Sustained containment is likely to be unmanageable. If those extra-terrestrial environments are capable of sustaining growth of terrestrial microorganisms, human travel and habitation will almost inevitably result in dissemination of those organisms that can grow and subsequently adapt and diversify from seeded organisms to the new environment.

**Figure 1:**
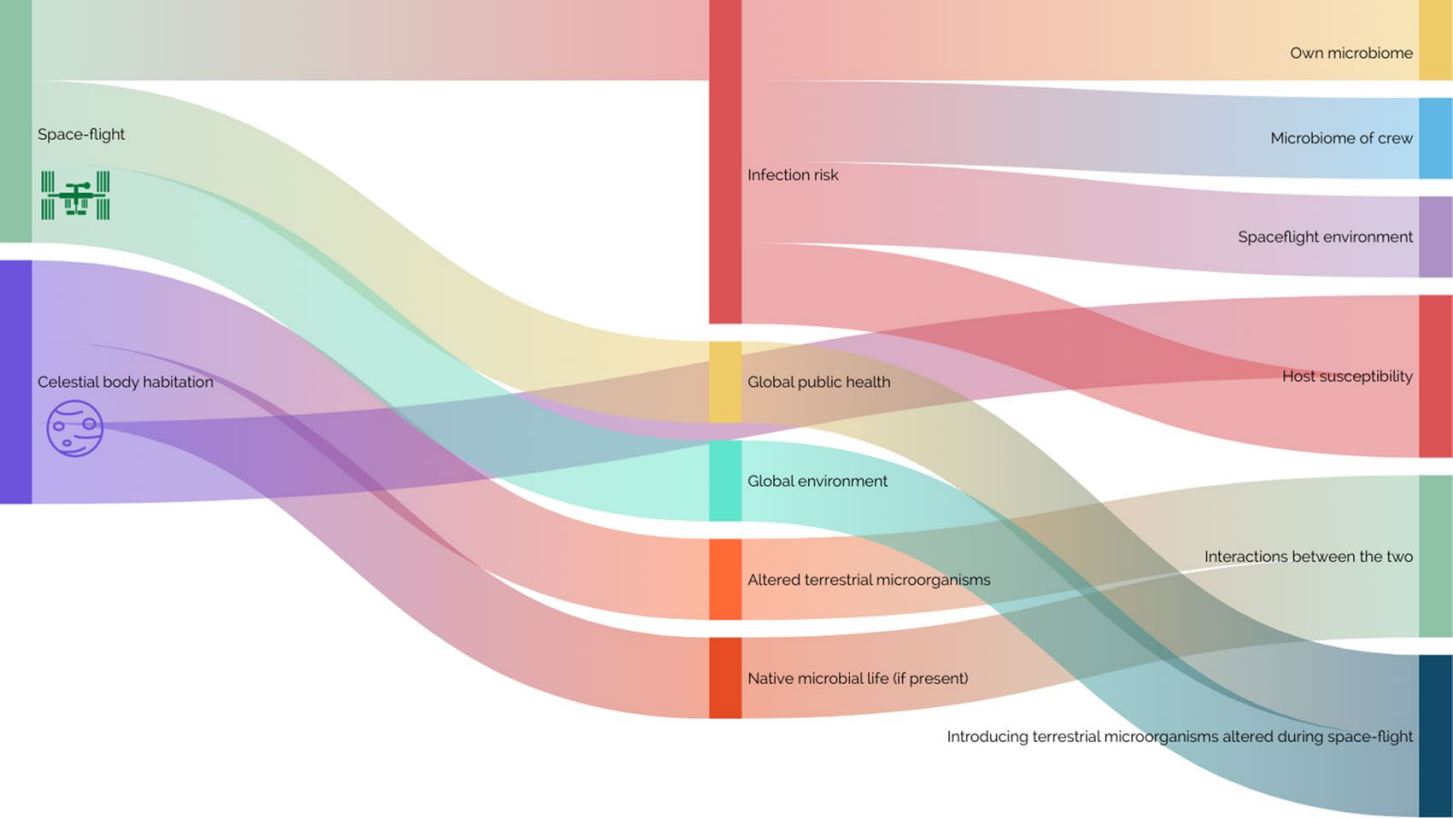
A sankey diagram visualising the key medical astro-microbiology considerations relating to spaceflight and celestial body habitation.

So far, our overall understanding of the microbiology and infectious disease related risks associated with a multi-planetary presence and prolonged spaceflight is very limited. The planned interconnectedness of Earth with orbital and celestial habitats requires public health considerations for both the outpost inhabitants and the terrestrial population alike. Our current understanding is guided by pioneering studies conducted mostly on the ISS or on microgravity and Mars simulating scenarios on Earth.^25,26^ Most terrestrial experiments have set limitations in their inability to simulate the type and amount of UV and cosmic ionising radiation encountered in space.^27^ This provides a background for review and interpretation of the literature in relation to microbiology and infection in the context of space travel and space settlement. This evidence base is growing but is currently limited. There overall appear to be the following two main considerations in relation to medical astro-microbiology: (i) space travel and (ii) celestial body outposts, each with unique risks and challenges (Fig. 1).

## Spaceflight

Spaceflight with our current technological capabilities exposes life (acellular, unicellular and multicellular) to three main physical stressors that differ from those encountered on Earth. These are microgravity, increased radiation (UV and cosmic/ionising)^MN4^

^**MN4**^High energy particles, originating from the Sun, travelling through space at near the speed of light (300,000 km/sec).

and circadian disruption. In this review we will focus on the effects of microgravity and radiation. The effects of spaceflight to circadian rhythm and its evident association with astronaut health and microbiomes has been studied and reviewed elsewhere.^28–30^ Other environmental factors such as space vacuum and thermal extremes are mitigated by our current spacecraft designs. Exposure of a host, in this case an astronaut, to a microbe may result in (1) failure for the organism to establish itself on the host, (2) colonisation of the host or (3) infection.^31^ The factors that determine the outcome are complex dynamic interactions dependent on the characteristics of the microbe, the host and other microbial elements of the host microbiome. The interaction may also contribute to heritable changes in a microbe that could influence its ability to transmit to and colonise or infect other hosts.^32^ A full understanding of the implications of space travel in relation to infection risk therefore requires an understanding of space travel associated effects on both human and microbial physiology. Furthermore, it could be speculated that stressors of spaceflight could cause an alteration to the incubation period of infectious diseases, though such effects are currently unmapped.

Throughout the over 20 years of permanent human presence in low Earth orbit, the effects of spaceflight to humans have been relatively well documented.^33,34^ A typical ISS expedition lasts about 6 months, although much longer stays in space have been recorded such as the one achieved by astronaut Scott Kelly and cosmonaut Mikhail Kornienko who spent 340 days in low-Earth orbit with scientists performing medical experiments (e.g., NASA's twins study). They launched on 27 March 2015 on Soyuz TMA-16M along with Gennady Padalka. The longest recorded stay in low-Earth orbit has been achieved by cosmonaut Valery Polyakov who remained in spaceflight for 438 days between 1994–1995.^35^ This compares to a 3-day duration for an one-way flight to the Moon and a 9-month duration for an one way flight to Mars.^36–38^ Beyond that timeframe, the effects of spaceflight on human physiology are unknown and therefore for longer flights to desirable locations with settlement prospects such as Jupiter’s moon Europa (628.3 million km distance, ~3 years of spaceflight) a better understanding of spaceflight associated effects to humans is required. By contrast, the effects of spaceflight on microbial life and the host microbiome have been studied and understood to a lesser extent.

### Host Susceptibility During Spaceflight

Spaceflight has been observed to induce alterations to human physiological functions for a variety of different systems. The immune and musculoskeletal systems appear to be the most susceptible to the stressors induced by spaceflight.^39,40^ A comprehensive review on the effects of spaceflight on the human body has been published previously.^41^ Due to its direct involvement in immunity and infection we will focus on the immune system. An overall dysregulation of the immune system in individuals undergoing spaceflight has been observed to include altered function and dispersion of different components including cytokines.^42^ Both the bone marrow and thymus are adversely affected by microgravity and space radiation with an overall impairment of lymphopoiesis^MN5^

^**MN5**^The processes of lymphocyte generation. B-cell lymphopoiesis takes place in the bone marrow, whereas T-cell lymphopoiesis takes place in the thymus.

which could lead to a disturbance of acquired immunity.^43^ Studies have also identified an increased incidence rate of hypersensitivities in crew members on board the ISS which may partially be a result of immune system impairment, possibly through a reported Th2 cytokine^MN6^

^**MN6**^Th2 type cytokines involve interleukins (IL) 4,5,13. Their main role is to promote immunoglobulin E and eosinophile responses.

sensitisation.^44^ Rashes are the most reported clinical events on board the ISS with an incidence rate of over 25-fold higher than that reported on Earth.^45^ Diagnoses also include eczema, psoriasis and contact dermatitis amongst others, with antihistamines being one of the most prescribed in-flight medications.^46^ The observed immune system dysregulations are also known to be linked with latent viral reactivation during spaceflight. A recent observation relates to a link between an increased level of plasma cytokines in astronauts and a high incidence rate of latent *Herpesviridae* reactivation.^47^ Several incidents of *Herpesviridae* reactivation amongst the ISS crew have already been reported.^48^ It is important to note that every study conducted so far, agrees that the immune alterations during spaceflight are acute, and they only persist for the duration of the mission. Of note, most of the studies assessing the effects of spaceflight on the immune system have been conducted using rodent models. The exact extent of the effects spaceflight conditions has on the human immune system is not fully understood at this time.

### The Closed Habitat Microbiome

Spaceflight by humans (or animals) currently occurs in hermetically sealed systems such as the ISS. This environment cannot be maintained as sterile because humans are hosts to a diverse microbiome that accompanies space travellers.^49^ Each traveller has a distinctive microbiome and therefore introduces new elements of the terrestrial microbiome into the ISS environment.^50^ Nevertheless, the diversity of microbes in the ISS is narrow compared to the microbial diversity of non-sealed terrestrial environments.^51^ This relatively narrow microbiome base is subject to selective pressures that differ in several aspects from those prevailing in terrestrial environments. The impacts of microgravity and of increased radiation (UV and ionising) exposure are widely recognised and often considered to predominate.^52,53^ However, there are other factors that may be equally significant in shaping change in organisms of terrestrial origin within space vehicles. Surface materials on which organisms grow as well as the composition of the atmosphere and the humidity of the spaceflight environment differ from many terrestrial environments in terms of quality and in lack of variation.^54^ The use of intensive surface cleaning and disinfection protocols are also likely to narrow the spacecraft microbiome and select for organisms with intrinsic or acquired resistance to the agents used.^55,56^ Lastly, the relative narrowness of the microbiome itself is likely to be a significant factor in shaping the microbiome from organisms that are introduced.

### Effect of Spaceflight to the Human Microbiome

The human microbiome has a widely recognised role in maintaining optimal human physiological functions. The microbiome relates to the compilation of different communities of bacteria, archaea, fungi and viruses whose composition is dynamic and influenced by environmental conditions.^57^ Disturbances to a healthy microbiome (dysbiosis) has been extensively linked with systemic diseases such as depression,^58^ Inflammatory Bowel Disease (IBD)^59^ and age-related neurological diseases such as Alzheimer’s disease amongst others.^60^ Gut and oral microbiome dysbiosis have also been extensively linked with an increased risk of infection including microbial translocation^MN7^

^**MN7**^The passage of bacterial cells or metabolites through across an anatomically intact intestinal barrier.

and bacteraemia^MN8^

^**MN8**^The presence of viable bacteria in the bloodstream..

^8,61,62^ Two well-established opportunistic infections caused by microbiome dysbiosis are these induced by *Clostridium dificile* (colitis) and *Candida albicans* (candidiasis).^63,64^ Additionally, the gut microbiome contributes to the maintenance of the intestinal wall. Gut dysbiosis can contribute to disruption of the gut barrier resulting in hyperpermeability of the gut walls or gut leakiness.^65^ This hyperpermeable state causes gastrointestinal (GI) tract dysfunction and can allow for translocation of both microbes and microbial products. Movement of microbes out of the gut and into systemic circulation has been associated with disorders including celiac disease.^66^ As with the human immune system, the human microbiome has been observed to be susceptible to space-related environmental stressors. A study conducted in a Hindlimb Unloading (HU), Dextran Sulphate Sodium (DSS) colitis induced mouse model in simulated microgravity conditions revealed an overall predisposition to colitis.^67^ When compared to the ground control mice, the mice exposed to microgravity had an associated early onset of colitis, a fourfold increase of segmented filamentous bacteria, more than a twofold decrease in regulatory T cells (T_reg_) absolute numbers as well as a reported twofold increase in colonic IL-1β and circulating neutrophils. The overall results of this and other studies utilising murine models^68^ suggest that both the rodent gut microbiome and the innate immune system, adversely respond to simulated microgravity contributing to a pro-inflammatory shift in the gut. The association of these findings with human physiology warrants further investigation. Studies examining the impact of spaceflight directly on the astronaut’s microbiome have also been attempted. A change in abundance of certain genera and species in the skin, nose, tongue and gastrointestinal tract of astronauts, which is usually a sign of dysbiosis, has been reported.^33,69^ Some signs of increased frequency of antimicrobial resistance (AMR) markers during spaceflight across five body areas including the skin and the mouth has been noted in one study.^33^ The AMR markers mostly related to tetracycline and erythromycin resistance. It needs to be highlighted that, so far human based microbiome studies during spaceflight have a very limited sample size (*n* = 4–9) and therefore statistical inference is difficult. An overall link between microbiome changes and the observed immune system impairment during spaceflight seems highly likely and larger studies are required when human space presence is extensive and larger cohorts are available. Furthermore, an association between the microbiome changes, impairment in immune system function and excessive hygiene could also be explored.

### The Hygiene Hypothesis

The original version of the hygiene hypothesis related to the suggestion that early-life exposure to infections prevents the development of allergies.^70^ The current description goes beyond that and includes non-pathogenic microorganisms (i.e., commensals and symbionts)^MN9^

^**MN9**^An organism that presents a long term biological interaction with another organism.

and expands to any inflammatory disease.^71^ A demonstration of the hygiene hypothesis relates to a significantly reduced risk for developing childhood asthma amongst children with early exposure to livestock in farm environments.^72^ Meta-analysis has shown an overall 25% reduction in asthma prevalence amongst children with early farm exposure.^73^ The reason behind this association is complex and it likely involves the effects of endotoxins, microbial diversity, and single pathogens. As mentioned before, pioneering astronaut-based microbiome studies have noted changes in both the skin and the gut microbiome amongst others, disturbances of which are known to be linked with inflammatory disease and skin pathologies. Microgravity, increased radiation (UV/ionising), stress and circadian rhythm disruptions associated with spaceflight could all be contributing to both the spaceflight associated microbiome dysbiosis and the immune dysregulation.^33^ Diet is generally well monitored during spaceflight, and it might be less of contributing factor to microbiome dysbiosis.^74^ A question that remains to be addressed relates to whether the highly clean environment of the ISS is another contributing factor to the observed microbiome changes and noted immune system dysregulation. Suggestions that an adoption of a more “natural” spaceflight environment could mitigate some of these health risks based on the hygiene hypothesis have been made previously and are well supported by literature.^75^ Given one of the main aims of space exploration is to identify microbial extra-terrestrial life, ultra-hygienic protocols during spaceflight have a rationale. Further research is required to elucidate the health benefits of a more “natural” spaceflight environment for the crew. A balance between crew’s health and “ultra- cleanliness” could then be achieved.

### Effect of Spaceflight on Microbial Species

Viral, bacterial and fungal agents are known to have their properties altered during spaceflight. Spaceflight conditions have been noted to have significant effects on microbial properties including a reported enhanced pathogenic ability for some bacterial and fungal species in in-vivo infection models. Overall, the best example we have in terms of understanding microbial properties in space relate to the experiments conducted throughout the over 20 years of the ISS presence in low Earth orbit. Although several studies have been conducted on viral, fungal and bacterial species there appears to be a notable lack of research on the adaptation of human disease associated parasites such as *Giardia lamblia* and *Cryptosporidium parvum* to spaceflight conditions.

#### Viruses

A review of our current understanding in spaceflight virology was recently published.^76^ So far, at least two studies using culture independent methods have included viruses aboard the ISS.^54,77^ NASA’s twin study provided a thorough assessment of gut microbiome changes associated with spaceflight capturing both the single-stranded  (ss) and double-stranded (ds) DNA virome.^77^ The twin study identified characteristic overall microbiome changes in composition and function associated with spaceflight. These changes could be due to isolation and dietary changes rather than spaceflight induced. Based on NASA’s shotgun metagenomics-based study EXTREMOPHILES, the ISS DNA virome^MN10^

^**MN10**^The total collection of DNA based bacterial and eukaryotic viruses identified in one biome.

appears to be dominated by bacteriophages (>95% of all viral reads).^54^ The remaining viral metagenomic reads belong to animal/human viruses. The most abundant DNA virus appeared to be the *Enterobacterales* bacteriophage *Microvirus* (68.84% relative abundance) followed by the bacteriophage family *Siphoviridae* (22.60% relative abundance). The most abundant non-bacteriophage related viral genera appeared to be *Mastadenovirus* which uses humans and other mammals as natural hosts (1.46% relative abundance). The human *Mastadenovirus* (HAdVs) is usually transmitted via the faecal–oral route and can cause a broad spectrum of diseases included pulmonary infection and gastroenteritis.^78^ Viruses of the family *Herpesviridae* were also identified including *Roseolivirus* (0.01% relative abundance) and *Varicellovirus* (0.09% relative abundance). Viruses of the genera *Roseolivrus* and *Varicellovirus* are well documented human pathogens and crucially can be transmitted by aerosolised respiratory droplets.^79,80^ There are two main limitations in studies utilising shotgun metagenomic sequencing for examining viruses: (i) most DNA shotgun metagenomics protocols target dsDNA and, therefore, ss-DNA viruses are excluded, and (ii) the absence of metatranscriptomic based studies which leads to a complete absence of information on RNA viruses. A mapping of the ISS ss-DNA and RNA virome would, therefore, be an important step in enhancing our ISS microbiome understanding. It has been suggested that the virome of closed environments such as buildings is largely originating from the outside environment with human presence strongly influencing the viral community of such spaces.^54,81^ For the ISS, the main sources of viruses are humans and equipment cross-contamination.

Furthermore, respiratory-based pathogen transmission during spaceflight requires special consideration and careful planning. In terms of airborne transmission of viruses in sealed systems, clarity is required in the use of the terms ‘droplets’ and ‘aerosols’, as these terms are sometimes used interchangeably. A scientific gold standard for an emission size cut-off between the two does not currently exist. Both the World Health Organisation (WHO) and the Centre for Disease Control (CDC) recommendations adopt a 5 μm cut-off to distinguish between the two categories.^82,83^ Emissions >5 μm in diameter are termed as droplets and the ones <5 μm in diameter are termed as aerosols. There are well-supported suggestions that this cut-off is not based on solid scientific evidence and is, therefore, misleading,^84^ but this is beyond the scope of this review. Aerosol deposition in human lungs is largely driven by gravitational sedimentation.^85^ Studies looking at the aerosol deposition of 0.5- to 3 μm particles in both microgravity and hypergravity^MN11^

^**MN11**^Hypergravity refers to an environment where the force of graivty is greater than that on the surface of the Earth (1 g).

during parabolic flights identified an increased level of aerosol deposition in human lungs under hypergravity conditions.^85^In microgravity conditions, the same study revealed that aerosols deposit peripherally (alveolar deposition) in the lung, beyond the protective effect of the mucociliary clearance system, increasing the risk for the establishment of a respiratory infection. Under normal conditions, respiratory droplets (>5 μm) deposit mainly in the upper and large airways.^86^ In microgravity-induced conditions (during parabolic flights), 5 μm diameter radiolabelled particles (resembling respiratory droplets) deposited in the central airways, where clearance mechanisms remain effective.^87^ Furthermore, the same study reported a reduction in 5 μm diameter particle deposition under microgravity conditions. Respiratory droplets are known to follow a gravity-influenced, semi-ballistic trajectory, and they usually settle no more than 2 meters from the source.^84^ It can, therefore, be hypothesized that microgravity would significantly alter the respiratory droplet trajectory, increasing its deposition range. Further studies are required to shed light into the fate of respiratory droplets under microgravity conditions as this is one of the main modes of human to human transmission for airborne bacteria and viruses.^84^ The effect of microgravity on the respiratory cilia and mucous production also remains unclear, alterations on which could also increase the risk of infection. Overall, in contrast with the immune system, the human respiratory system appears to remain unaffected in microgravity conditions and there is no known overall degradation in lung function.^88^ From what is known so far, an increased infection risk due to reduced lung function is not anticipated and the transmission risk relates to the effects of microgravity on aerosol transmission and deposition.

It is well documented that latent viruses, particularly of the family *Herpesviridae*, are commonly reactivated in astronauts on the ISS. This is suspected to be due to the overall decrease in immune activity as previously discussed.^89^ The viral shedding, determined as viral copies/salivary-mL, for three viruses of the family, *Herpesviridae*: Epstein–Barr virus (EBV), Varicella Zoster Virus (VZV) and Cytomegalovirus (CMV), is known to significantly increase during spaceflight.^17^ A linear relationship between duration of spaceflight and viral shedding (viral copy numbers) was also identified in the same study. A previous study targeting VZV showcased that saliva samples of the ISS crew contained live, infectious viral particles.^90^ Increased viral load in oral/respiratory secretions may cause an increased risk of transmission during prolonged spaceflight. All current studies on viral shedding during spaceflight are limited to a 6-month timeframe, due to the nature of spaceflight missions. The effects of prolonged spaceflight to viral shedding beyond the 6 months are, therefore, not documented. Furthermore, adaptive immune system function remains impaired during spaceflight of up to 6 months in duration, which could further contribute to increased viral shedding and an increasing risk of infection.^91^ A list of DNA viruses with known implications to medical astro-microbiology can be seen in Table 1. As a countermeasure to the above observations, it has been suggested that astronauts would need to be vaccinated against the VZV prior to spaceflight.^17^ The overall link between microgravity and viral shedding is yet unclear. Increased stress and reduced immunity during spaceflight are also anticipated to play a key role in latent viral reactivation in the ganglion, with saliva shedding to follow.^17^ Last, it can be hypothesized that viral shedding and transmissibility could be a lesser concern for Martian settlements due to Mars gravity (38% that of Earth’s gravity) mitigating the risk for viral reactivation in addition to reversing the effects of microgravity on adaptive immunity^MN9^. Further research is required in that regard.

**Table 1:** Summary of DNA viruses with a known medical astro-microbiology association.

Medical astro-microbiology DNA viruses of interest					
Species	Family	Considerations	Transmission	Clinical importance	References
Varicella-Zoster Virus (VZV)	*Herpesviridae*	1. Reactivation 2. Higher viral load 3. Higher risk of transmission	Direct contact Droplet/airborne	Chickenpox (more easily spread), shingles	^17,48^
Epstein-Barr virus (EBV)	*Herpesviridae*	1. Reactivation 2. Higher viral load 3. Higher risk of transmission due to microgravity	Direct contact	Infectious mononucleosis	^17,48^
Herpes simplex virus (HSV-1)	*Herpesviridae*	1. Reactivation, 2. Increased illness severity (microgravity)	Direct contact	Oral and genital herpes	^17,48^
Kaposi’s sarcoma-associated herpesvirus	*Herpesviridae*	1. Microgravity influences viral reactivation	Direct contact (Including sexual contact)	Kaposi's sarcoma (mainly in profoundly immunocompromised patients)	^93^

Overall, during human spaceflight there is a significant risk associated with airborne transmission of viruses. This is currently mitigated by NASA’s HSP aimed to reduce risk for contracting infectious disease during space travel through a combination of vaccination and a 14-day pre-flight quarantine. If space travel becomes accessible to more people and space settlements are established, the application of protocols such as the HSP may be very difficult to enforce and hence, up to date vaccinations for space travellers as well as other in-flight Infection Prevention and Control (IPC) protocols such as the availability of in-flight en-suite isolation rooms with negative pressure^**MN12**^

^**MN12**^Isolation rooms where that filter any air flowing out of the room to prevent airborne pathogen transmission.

could be beneficial for the mitigation of associated transmission risks.^92^

#### Bacteria

In contrast with the ISS virome, the ISS bacteriome^MN13^

^**MN13**^The total community of bacterial microorganisms residing in a particular environment.

is well documented and relatively well understood. A comprehensive review of bacterial virulence and antibiotic susceptibility has been previously conducted.^94^ A key consideration relates to bacterial behaviour in space. Certain bacteria have been reported to demonstrate changes associated with an increase in virulence under spaceflight conditions, based on assessments in in-vivo infection models. This change in bacterial virulence could partly be due to the effects of global regulators (GRs). GRs are transcription factors that coordinate responses to environmental stressors by coordinating the expression of thousands of genes.^95^ Other studies of bacteria have reported a change or a decrease in markers associated with virulence properties.^96^ Pivotal to the characterisation of the bacterial diversity on board the ISS have been the Microbial Tracking (MT) 1–3 studies.

Initial culture-independent metataxonomic analysis of the ISS filter debris, using 16S rRNA gene amplicon sequencing, revealed an ISS associated bacteriome dominated by the phyla *Actinobacteria* (69% relative abundance) and *Firmicutes* (29.63% relative abundance) with the genera *Corynebacterium* (phylum *Actinobacteria*—67.3% relative abundance) and *Streptococcus* (phylum *Firmicutes*—20.46% relative abundance) dominating the pyrosequences.^25^ In parallel, culture-dependant analysis in the same study revealed that from the culturable species, those of the genera *Bacillus* and *Staphylococcus* were the most isolated. An absence of reads associated with spore-forming species during the use of metataxonomics^MN14^

^**MN14**^The sequencing of target regions of the bacterial ribosomal RNA (rRNA) with the aim of taxonomically characterising a microbial community.

was noted, attributed to the difficulty of extracting DNA from spore-forming species along with a possible association between microgravity and enhanced hardness of bacterial spores.^25^ Subsequent shotgun metagenomics analysis identified that the ISS surface bacteriome was dominated by species associated with the human skin, mainly those of *Staphylococcus.* This verified the culture-dependent findings of previous studies.^97^ In relation to culture independent approaches, most protocols used during DNA extraction can introduce bias which can distort the true abundance of taxa and species based on differences between the taxa such as DNA yield.^98^ Overall, *Staphylococcus epidermidis* was the most predominant bacterial species followed by *Cutibacterium acnes*, another human skin-associated commensal. These species can be associated with opportunistic infection in certain human cohorts with weakened immune system and both are associated with AMR in clinical settings.^99–101^ The overall risk of infection, however, is considered low. Neither of these two species have previously been associated with astronaut infections during spaceflight. A preliminary examination of the ISS resistome reveals that resistance genes associated with macrolide-lincosamide-streptogramin (MLS) antibiotics are the predominant Antimicrobial Resistance Genes (ARGs) identified; a resistance pattern commonly encountered in skin-associated species such as *C. acnes*.^99,102^

Amongst all the studies conducted so far, some bacterial species appear to play an enhanced role in the ISS related bacteriome:

##### ***Bacilllus cereus*** sensu lato (s.l.)

Spore forming bacteria have always been a concern from a planetary protection context due to their ability to cause human disease while withstanding disinfection protocols and displaying resistance to the harsh environments of space. One of the most abundant spore-forming species aboard the ISS are these of the *Bacillus cereus* s.l. group. A group of 11 non-toxin-producing *Bacillus cereus* strains were recently identified from ISS surfaces and extensively studied.^103^ These strains displayed close genomic relatedness to *Bacillus anthracis* and are, therefore, grouped to a distinct *B. anthracis* clade. In addition, these strains characteristically lacked the plasmids (pXO1, pXO2) associated with *B. anthracis* virulence.

Pathogenetically, key components of virulence in *B. cereus* and *Bacillus thuringiensis* were also missing, and overall clinical characteristics of this *B. anthracis* clade requires further studies. Several strains of *Bacillus amyloliquefaciens* (*n* = 9) and *B. thuringiensis* (*n* = 10) have also been isolated on consecutive days from surfaces on the ISS.^104^ Of note, the *B. thuringiensis* strains have been shown to possess an array of virulence factors associated with enteropathogenicity^MN15^

^**MN15**^Disease of the intestinal tract.

(e.g., non-hemolytic enterotoxin) and the siderophore petrobactin associated with virulence in *B. anthracis*.^105,106^ Some of the ISS originating *B. thuringiensis* strains are phenotypically resistant to carbapenems; a type of resistance rarely reported in *Bacillus* species.^107^ It is important to mention that non-toxigenic *B. anthracis* along with most *B. cereus* s.l. species are ubiquitously present on Earth and are rarely pathogenic. Further studies are required to fully characterize the medical astro-microbiology implications and any possible health risks associated with this genus during spaceflight.

##### ***Kalamiella piersonii***

*Kalamiella* is a novel genus in the *Enterobacterales* order that currently consists of a single species,* K. piersonii*.^108^ *Kalamiella* is the first bacterial genus to be identified outside of the terrestrial environment. Culture independent and machine learning analysis revealed *K. piersonii* to be highly prevalent in the ISS microbiome and dominating the ISS resistome^MN16^

^**MN16**^The total collection of ARGs in a microbiome..

^109^ Several strains of *K. piersonii* (*n* = 33) have been isolated from surfaces of the ISS, dating back to 2016, rendering *K. piersonii* an organism of interest for medical astro-microbiology. Initial analysis predicted *K. piersonii* as a non-pathogenic species with no human association. Since its first report in 2019, a few clinical cases of *K. piersonii* have been reported in humans including bacteraemia, meningitis and kidney stones disease amongst others.^110,111^ Preliminary genomic analysis of this novel species has identified that all strains examined so far exhibit a multidrug resistant (MDR) phenotype and genotype along with a hypervirulence associated (Hv) genotype. The Hv genotype relates to the presence of a 262 Kbp non mobilizable plasmid encoding for the aerobactin gene cluster (*iucA-D/iutA*) along with the vibriobactin utilisation protein (*viuB*) and the virulence transcriptional regulator (*virB*).^112^ Drug resistance to cephalosporins, quinolones, aminopenicillins, rifampicin, piperacillin and glycopeptides has already been reported.^110,111^ Resistance to last resort carbapenem, tigecycline and polymyxin antibiotics has not been reported for any of the *K. piersonii* strains to date. Given the persistence of *K. piersonii* on board the ISS, its potential to cause a variety of different infections and its MDR status, further investigations are required to clarify the pathogenicity of this novel human pathogen both in terrestrial and spaceflight settings.

##### ***Klebsiella pneumoniae***

*K. pneumoniae* is a well-characterized human commensal and pathogen, one associated with hypervirulence and acquired AMR to last resort antimicrobial agents. The World Health Organisation (WHO) has categorised carbapenem-resistant *K. pneumoniae* (CRKP) as a priority 1 (critical) pathogen for the development of new antibiotics.^113^ CRKP infections have a reported pooled mortality rate of 42.14% vs 21.16% observed for carbapenem-susceptible *K. pneumoniae* (CSKP) associated bloodstream infections.^114^ *Klebsiella* species are known members of the human gut microbiome so their presence on board the ISS is expected. Indeed, several *Klebsiella* isolates have been identified from ISS surfaces using culture-dependent methods.^115^ Interestingly, a recent study on the metabolic modelling of the ISS microbiome revealed *K. pneumoniae* as a having a key role in metabolic interactions between microorganisms.^18^ In particular*, K. pneumoniae* appeared to be exceptionally beneficial for the survival of *Pantoea* species. Of clinical relevance in terms of polymicrobial infections^MN17^

^**MN17**^Diseases caused by any combination of viral, bacterial and fungal pathogens.,

in actual spaceflight conditions *K. pneumoniae* appears to be antagonistic (parasitic) against filamentous fungi of the genus *Aspergillus* in agreement with terrestrial observations.^116^ Given the clinical relevance and apparent metabolic importance of *K. pneumoniae* in spaceflight conditions, a complete genomic characterisation of the virulence and drug resistance of the identified *K. pneumoniae* strains would be beneficial, along with an extended effort of mapping its synergistic and antagonistic interactions in terms of the ISS microbiome.

##### ***Enterobacter bugadensis***

*E. bugandensis* is a recently identified species of *Enterobacter*. It is associated with cases of sepsis in neonates and immunocompromised patients.^117^ Multidrug and metal-resistant strains of *E. bugandensis* (*n* = 5) have been isolated from the ISS and presented a close genetic resemblance with the clinically isolated strains EB-247 and 153-ECLO.^118^ All strains appeared resistant to cefazolin, cefoxitin, oxacillin, penicillin, and rifampicin, while a subset were non-susceptible to ciprofloxacin and erythromycin. Erythromycin is not clinically used to treat *Enterobacter* species infections, whereas ciprofloxacin is an antibiotic that is used for clinical cases of infections caused by *Enterobacter*. Resistance to penicillin, cefoxitin and cefazolin is intrinsic in *Enterobacter* species due to the production of the AmpC beta lactamase.^119^ The multiple antibiotic resistance (MAR) locus was also reported as a main mediator of resistance in these strains. High levels of MDR^MN18^

^**MN18**^Phenotypic non-susceptibility of a bacterial microorganism in at least 1 antibiotic agent from at least 3 different classes of antibiotics.

in *E. bugandensis* was recently observed in clinical strains.^117^ Clinical options for treatment of such infections in space conditions could be challenging with carbapenems being the antibiotic of choice in that instance.^117,120^ Only ertapenem is currently available as a carbapenem treatment option on board the ISS.

##### ***Escherichia coli***

*E. coli* is another member of the order *Enterobacterales* and as well as a characterised human intestinal commensal. Apart from its scientific benefit, it can also be a versatile human pathogen.^121^*E. coli* strains that are associated with human pathogenesis are broadly separated between intestinal pathogenic *E. coli* (IPEC) or extraintestinal pathogenic *E. coli* (ExPEC). Aside from its pathogen potential, *E. coli* strains are known to have a great capacity to accumulate ARGs via horizontal gene transfer with MDR (e.g. ST131) and Extended Drug Resistant (XDR) lineages (e.g. ST361) described so far.^122,123^Given its ubiquitous dissemination and association with AMR, the effects of microgravity on *E. coli’s* resistance properties have been a focus of NASA’s autonomous *E. coli* AntiMicrobial Satellite (EcAMSat) mission. The experiment exposed two strains of AMG1 uropathogenic *E. coli* (UPEC), a wild type (WT) strain and a strain with a deleted *rpoS* gene (∆rpoS) (stress response regulator) to spaceflight conditions.^124^ All strains were grown to stationary phase at 37 °C and tested with three different doses of the antibiotic gentamycin. A parallel ground control experiment was also maintained. The findings suggested that the microgravity exposed strains were more susceptible to gentamycin compared to the ground control strains. The spaceflight strains also exhibited a slower metabolism, with the ∆rpoS mutant being 34–37% less metabolically active compared to the WT.^125^ The EcAMSat study concluded that the *rpoS* gene and its downstream products might be good therapeutic targets for treating *E. coli* induced infections in space and on Earth alike. Other studies exposing *E. coli* strains to different gentamycin concentrations in spaceflight conditions on board the ISS have presented contradicting results. A 2018 study showed that within 49 h of being cultured, a non-pathogenic *E. coli* strain ATCC-4157, was able to grow to a higher concentration of gentamycin in spaceflight conditions (>175 μg/mL) compared to the Earthly control (75 μg/mL).^126^ Additionally, 50 stress-response regulated genes were observed to be upregulated in spaceflight conditions, providing further evidence of the importance of stress-response for *E. coli’s* spaceflight adaptation. Other experiments conducted on ISS utilising the same ATCC-4157 strain verified the ability of the strain to grow to a higher concentration of gentamicin (>175μg/mL) compared to the Earthly control (75μg/mL).^127^ Overall, as with other *Enterobacterales*,^MN19^

^**MN19**^Enterobacerales is an order of Gram negative bacteria that are commonly found in the gastrointestinal tract of human and animals. Many common human pathogens belong to this order.

a strain-specific response to space conditions is observed for *E. coli*. As with other *Enterobacterales* experiments, an overall increase in experimentation with well-characterised clinical strains could aid in elucidating the clinical relevance of these findings. An overview of bacterial species of medical astro-microbiology interest based on our current understanding can be seen in Table 2.

**Table 2:** Summary of bacterial pathogens with known medical astro-microbiology association.

Medical astro-microbiology bacterial pathogens of interest					
Species	Space related observation	Biofilm forming	Transmission	Clinical importance	References
*Salmonella typhimurium*	Enhanced virulence	Yes	Direct contact	Gastrointestinal infections	^128^
*Staphylococcus warneri*	Enhanced virulence	Yes	Direct contact	Rare opportunistic pathogen (UTI)	
*Serratia marcescens*	Enhanced virulence	Yes	Direct contact	Broad (urinary tract infections, wound infections, respiratory infections)	^19^
*Enterobacter bugandensis*	Multidrug resistance	Yes	Direct contact	Severe neonatal and immunocompromised patient infections (sepsis)	^118^
*Klebsiella pneumoniae*	Drug resistance, metabolic importance	Yes	Direct contact	Broad (pneumonia, bloodstream infections, meningitis)	^18^
*Kalamiella piersonii*	Persistence, multidrug resistance, hypervirulence	Yes	Unknown	Broad (wound infection, sepsis, meningitis)	^112,129^
*Bacillus spp.*	Spore forming, persistence, carbapenem resistance, virulence	Yes	Direct contact	Foodborne illness, gastrointestinal infections	^103^

Some of the opportunistic pathogens that have undergone exposure to spaceflight or microgravity simulated conditions have been observed to have enhanced pathogenic properties in in-vivo models though the clinical significance of these findings require further investigation. While virulence was increased while under the influence of spaceflight conditions, any increase that was observed has been noted to decrease to pre-flight levels upon a return to Earth.^19^

#### Bacterial Pathogens with Evidence of Enhanced Virulence in Spaceflight Conditions

The most well-documented example of microgravity-associated enhancement of virulence relates to *Salmonella enterica* SSalmonella enterica serovar Typhimurium *S. typhimurium* is a zoonotic pathogen that is commonly associated with self-limiting gastroenteritis in humans and occasionally associated with blood stream infections. Enhanced virulence for *S. typhimurium* has been reported in murine infection models^MN20^

^**MN20**^A mice based infection model commonly used to study infection of human pathogens and treatment responses.

during spaceflight.^128,130^ The enhanced virulence was identified based on the observation of a reduction in LD_50_ value and an increase in both percent mortality and time to death from point of infection for mice infected with spaceflight associated cultures compared to ground control cultures. This enhanced virulence was likely regulated by the environmental response regulator^MN21^

^**MN21**^Genes that are responsive to environmental exposures which they subsequently upregulate or downregulate the expression of specific gene clusters.

RNA-binding protein Hfq.^128^ These findings were further supported by a study conducted on *Salmonella enterica* Serovar Enteritidis during spaceflight (1 month duration). The study revealed that spaceflight was associated with an enhanced growth rate compared to the ground control. Differentially expressed proteins associated with energy production and transmembrane transport were also reported.^131^ Furthermore, the same study identified that the microgravity induced downregulation of the *oppA* gene, a member of the oligopeptide transport system OPP, leading to amikacin resistance. The opportunistic pathogen *Serratia marcescens* was also shown to undergo spaceflight-induced changes associated with enhanced virulence as this was evaluated in a *Drosophila melanogaster* infection model. The reported virulence associated changes were proposed to be microgravity associated.^19^ *S. marcescens* is a pathogen with increasing rates of drug resistance amongst clinical isolates and is commonly associated with nosocomial infections including bacteraemia.^132^ The Gram-positive skin commensal *Staphylococcus warneri* was also observed to rapidly adapt to microgravity and radiation conditions during prolonged spaceflight (79 days duration). Notable changes included an increase in its biofilm formation ability, while subtle changes to the genome (15 InDels) and phenome (cell wall hardness and chemical sensitivity) were also observed.^133^ *S. warneri* as most other coagulase-negative staphylococci is rarely associated with infections in healthy individuals and is mostly associated with infection (e.g. bacteraemia) in individuals with prosthetic devices.^134,135^

Selective pressure imposed by microgravity exposure is currently hypothesised to be the predominant causative agent for the observed enhancement of pathogenic properties in some bacterial species. Microgravity has been shown to have influence on microbial properties including enhancing stress resistance of bacteria.^136^ The exact mechanism behind these effects is currently not known. Due to the difficulty of conducting experiments in spaceflight conditions, terrestrial simulated microgravity models are often utilised, in specific low shear modelled microgravity (LSMMG)^MN22^

^**MN22**^A technique utilised to simulate microgravity on a terrestrial environment.

simulations.

However, not all pathogens undergo an enhancement of virulence when exposed to microgravity conditions.

#### Bacterial Pathogens with Evidence of Reduced Virulence in Spaceflight Conditions

Methicillin-resistant *Staphylococcus aureus* (MRSA), *Listeria monocytogenes* and *Enterococcus faecalis* are all pathogens that have been observed to have a reduction in virulence on Earth based spaceflight analog models.^137^ A thorough study focused on the in-vivo virulence of *Klebsiella pneumoniae*, *Pseudomonas aeruginosa* and *Proteus mirabilis* strains in larval and adult worm models under spaceflight conditions. Overall, spaceflight appeared to reduce the virulence of *Klebsiella pneumoniae* strain ATCC-8052, whereas the virulence of *Proteus mirabilis* strain ATCC-4630 and *Pseudomonas aeruginosa* strain ATCC-BAA-47 remained unaffected.^138^ *Acinetobacter baumanii* and *Acinetobacter schindleri* are human opportunistic pathogens often associated with Hospital Acquired Infections (HAIs) and have both been shown to have decreased biofilm formation abilities after spaceflight on the Shenzoun 10 and 11 spacecrafts commissioned by China National Space Administration (CNSA).^139,140^ To confirm these findigs, coherent international effors are required. Experiments in LSMMG conditions for *Yersinia pestis* (the causative agent of bubonic, septicaemic, and pneumonic plague) also reported a decreased virulence potential as this was evaluated in cell culture infection assays.^141^ For most of these experiments, strains from the American Tissue Type Collection (ATCC)^MN23^

^**MN23**^A standardised collection of reference microorganisms used for research and development.

**Figure 2:**
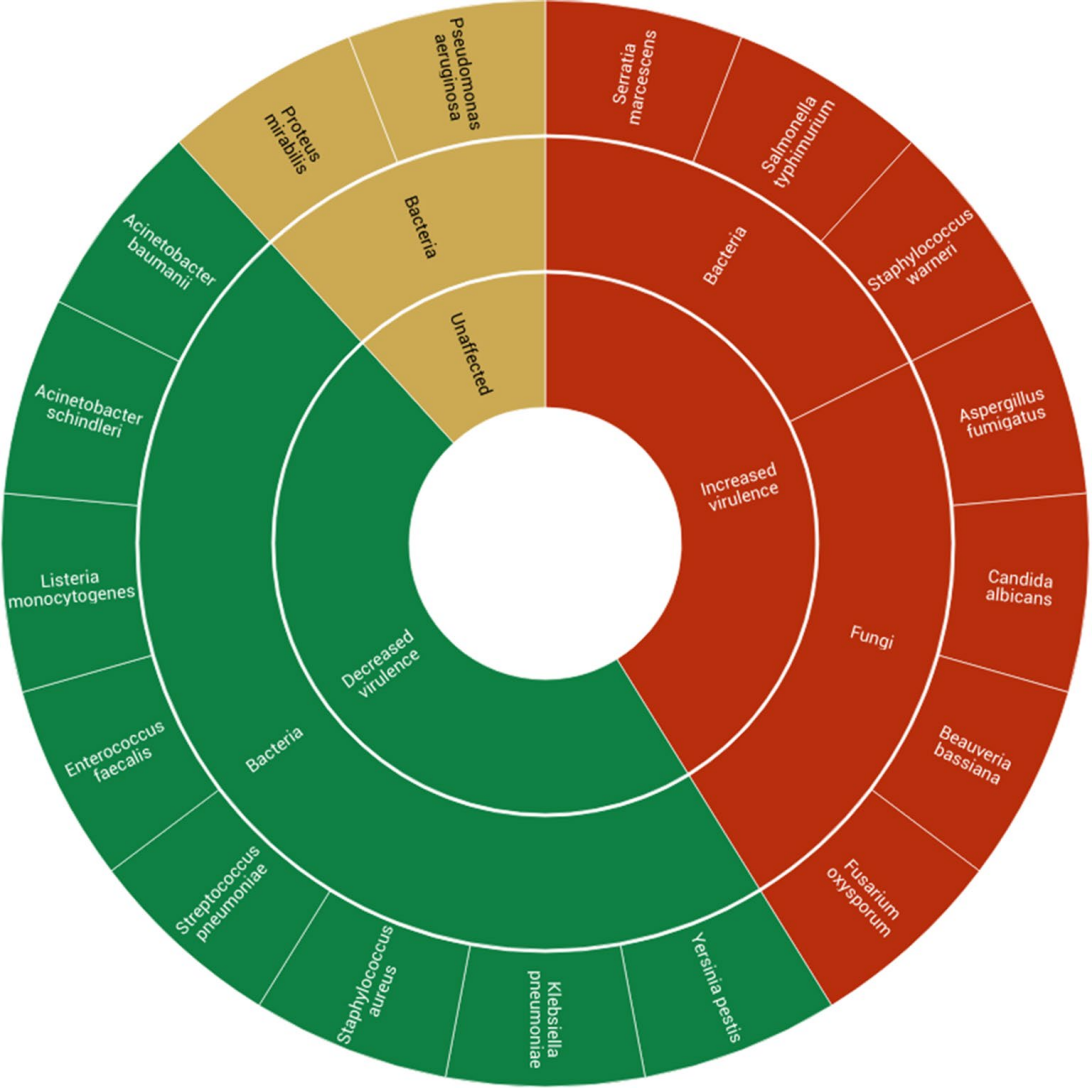
Starburst showing bacterial species (*n* = 13) and fungal species (*n* = 4) with experimentally confirmed altered virulence properties under microgravity conditions.

were selected. Future experiments could utilise clinical strains with a demonstrated hypervirulence phenotype and genotype such as strains of Hypervirulent *K. pneumoniae* (hvKp). Documenting the effects of microgravity and radiation (UV, ionising) on hypervirulent strains would clarify the clinical significance of the reported reduced virulence findings. A summary of bacterial and fungal pathogens with evidence of increased virulence during spaceflight can be seen in Fig. 2.

#### Antimicrobial Resistance (AMR)

Overall, AMR can be seen as an ecological consequence of the widespread use of antibiotics. Antibiotic-resistant organisms (AROs) are frequently encountered in healthcare settings where antibiotic selective pressure is intensive. AMR is often referred to as a ‘silent pandemic’. The effects of spaceflight conditions on AMR appears to be species and even strain specific, although it is evident that most species very rapidly adapt to antibiotic exposure in simulated microgravity conditions using LSMMG apparatus. Further studies could clarify if AMR associated adaptability is different in actual microgravity conditions and Earth. In contrast with Earth, treatment options during spaceflight are very limited and medical interventions are vastly more complicated.^94^ Taking this into account, antibiotic usage and AMR dissemination in spaceflight needs to be closely monitored.

Isolates of *Enterobacter* found on ISS surfaces have been identified to possess the metallo-beta-lactamase encoding *IMP-2* gene that is known to confer resistance to carbapenems, clinically last resort antibiotics reserved for treatment of complicated infections.^142^ Phenotypic resistant to carbapenems (ertapenem and meropenem) for ISS originating strains of *Bacillus* has also recently been identified, with current efforts focussing on elucidating whether its intrinsic or acquired (ongoing research). *E. coli* has also been shown to rapidly adapt to LSMMG conditions with emerging resistance to cefalotin, cefuroxime, cefuroxime axetil, cefoxitin, and tetracycline after 1000 bacterial generations.^143^ The isogenic strains of this experiment were grown in high-aspect-ratio vessels (HARVs) intermittently exposed to chloramphenicol between cycles to prevent contamination of the samples. Resistance in *E. coli* appeared to be caused by a combination of increased mutagenesis along with three transposon mediated rearrangements. The same study when repeated with the intermittent use of steam for sterilization instead of chloramphenicol did not result in acquired antibiotic resistance.^144^ Overall, the findings of these studies suggest that simulated microgravity conditions do not appear to reduce the antibiotic stress adaptation for the tested *E. coli* strains.

The most common method of dissemination of antimicrobial resistance genes (ARGs) is through mobile genetic elements (MGEs).^MN24^

^**MN24**^Segments of genetic material that can move within a genome and/or can be transferred to other bacterial cells.

The ISS and the Antarctic Research Station Concordia (ARSC) are both confined and secluded built environments that experience extreme conditions with the ARSC being used as a model environment for space travel.^145^ The major difference between the two habitats is the inclusion of radiation and microgravity as environmental pressures aboard the ISS. A study has been conducted comparing AMR and the conjugative transfer capacity of *Staphylococcus* and *Enterococcus* isolates on the ISS and in the ARSC. In this study, the ISS strains showed a higher abundance of ARG carriage, compared to the ARSC strains.^146^ Interestingly, the ISS strains were also shown to possess higher horizontal gene transfer (HGT)^MN25^

^**MN25**^Asexual movement of genetic information between genomes of the same or different species.

capacity via the presence of virulence regulatory genes (*vir*). Virulence regulatory genes have been extensively identified in the accessory genome of ISS strains and could be tested as markers for microgravity induced virulence.

Studies using culture independent methods looking at ISS surfaces have revealed a complex ISS resistome, with more than 123 ARGs identified, with those for β-lactam and trimethoprim resistance identified as the most prevalent.^142^ HGT abilities measured in both the ISS and in the LSMMG scenarios on Earth have revealed species dependant changes, with the *Bacillus* species possibly showcasing an increased plasmid transfer efficiency.^147^ Apart from *Bacillus* species the overall HGT rate for other Gram-negative bacteria tested in microgravity conditions remained unaffected.^148,149^ A recent study using *S. aureus* strains clearly demonstrated that simulated microgravity directly promotes HGT of ARGs even in the absence of antibiotic selective pressure.^150^ The overall pattern seen for AMR and HGT under the influence of microgravity is the same as that seen for virulence: highly variable and species specific. Overall, it is evident that a one-fits-all approach is not adequate to map the effects of microgravity and overall spaceflight on AMR and the dissemination of ARGs. Therefore, a coordinated effort is required to understand, document and map these effects in more clinically relevant species and strains.

#### Fungi

Similar to the absence of a universal effect of spaceflight-related environmental factors on the pathogenic properties of bacteria, there is no universal positive or negative effect of spaceflight on fungi. Overall, filamentous fungi appear well adapted to spaceflight conditions. Metataxonomic analysis of ISS surfaces utilising the fungal Internal Transcribed Spacer (ITS) region, revealed a fungal population comprised of one phylum and eleven genera^151^ The culture-dependent methods employed by the same study identified 81 cultivatable fungal isolates which were dominated by *Rhodotorula mucilaginosa* (41% of the isolates) and *Penicillium chrysogenum* (15% of the isolates).

Analysis conducted for two *Aspergillus fumigatus* strains isolated from air and surfaces of the ISS identified that the ISS strains exhibited enhanced lethality in a neutrophil-deficient larval zebrafish model of invasive aspergillosis compared to clinical isolates (Af293 and CEA10).^152,153^ *Aspergillus fumigatus* has also been isolated from HEPA filters aboard the ISS as part of the Microbial Observatory Experiments. ISS *A. fumigatus* strains presented an increased abundance of proteins associated with stress responses (Pst2, ArtA) and carbohydrate and secondary metabolism (PdcA, AcuE).^26^ An increase in toxin production (Asp-hemolysin) was also reported in the same study, hinting to a potential increase in virulence. Indeed, a follow-up analysis on ISS *A. fumigatus* strains utilising larval zebrafish virulence assays reported a strain-specific increase in secondary metabolites (SMs) related to radiation (UV-C) protection and enhanced virulence.^154^ The human commensal and opportunistic pathogen *Candida albicans* was also shown to exhibit an increase in both virulence and resistance against the antifungal agent amphotericin-B during spaceflight conditions.^155^ A study conducted on the ChangZheng 5 space shuttle on the entomopathogenic fungus, *Beauveria bassiana*, revealed a series of SMs (toxins) produced exclusively by the spaceflight associated strains, with an overall insecticidal activity.^138,156^ As part of the “Veggie” project (an ISS based project for studying the effect of plant growth and function in spaceflight conditions), the *Zinnia hybrida* plants utilised in the study became infected with the widespread plant and opportunistic human fungal pathogen *Fusarium oxysporum*.^157^ Two *F. oxysporum* isolates were recovered from the infected plants and genomic analysis revealed a close genetic relatedness with strains isolated following the Chernobyl disaster as well as a clinical strain (FOSC 3-a) from a blood culture of a patient with fusariosis. *F. oxysporum* strains have also been isolated from the dining table of the ISS.^158^ These strains were studied in a context of a virulence assay using an immunocompromised *Caenorhabditis elegans* model of fusariosis^MN26^

^**MN26**^A fungal infection from pathogens of the genus *Fusarium* affecting human, plants and animals..

Both strains appeared capable of establishing an infection, with one strain (ISS-F40) showing enhanced virulence abilities via hypha piercing and other mechanisms.

*Aspergillus nidulans* strains have also been characterised for genomic, proteomic and metabolomic changes under spaceflight conditions.^159^ The ISS grown *A. nidulans* samples showed positive selection^MN27^

^**MN27**^The tendency of traits to become more prevalent in a bacterial population.

for a small subset of protein coding genes (*n* = 5). Interestingly, genomic indications of variations in transposable element activity were also noted under spaceflight conditions as reported for the bacteria of the species *S. aureus*.^160^ Overall, through heterogeneous genomic expression, *A. nidulans* has been proposed as a host capable for the production of secondary metabolites with antibiotic activity during prolonged spaceflight in deep space, which can be used in case spacecraft therapeutic stocks are exhausted. Another species of *Aspergillus* that has been isolated and studied from on board the ISS is that of *Aspergillus niger. A. niger* is a ubiquitous fungus that thrives in environments like the ISS. Initial metabolomic based analyses of the *A. niger* ISS isolated strain JSC-093350089 reported an increased production of naphtho-γ-pyrone along with potentially therapeutically relevant secondary metabolites including the antioxidant pyranonigrin A.^154,161^ Subsequent experiments comparing the same *A. niger* exposed to the ISS conditions against a ground control revealed that ISS conditions permanently altered the strains metabolic functions with a significant enhancement in production of the pyranonigrin A.^162^ A summary of fungal pathogens with medical astro-microbiology association can be seen in Table 3.

**Table 3:** Summary of fungal pathogens with known medical astro-microbiology association.

Medical astro-microbiology fungal pathogens of interest					
Species	Space related observation	Human pathogen	Transmission	Clinical importance	References
*Aspergillus fumigatus*	Enhanced virulence	Yes	Droplet/airborne	Aspergillosis (Immunocompromised or chronic lung conditions)	^152^
*Candida albicans*	Enhanced virulence, Antifungal resistance	Yes	Direct contact	Candidiasis (usually vaginal yeast infection)	^155^
*Beauveria bassiana*	Enhanced virulence	Very rarely	Direct contact, droplet/airborne	Disseminated infection	^156^
*Fusarium oxysporum*	Enhanced virulence	Yes	Direct contact	Broad (onychomycosis, keratitis, skin infection, pulmonary infection)	^158,163^

### Global Public Health and the Global Environment

**Figure 3:**
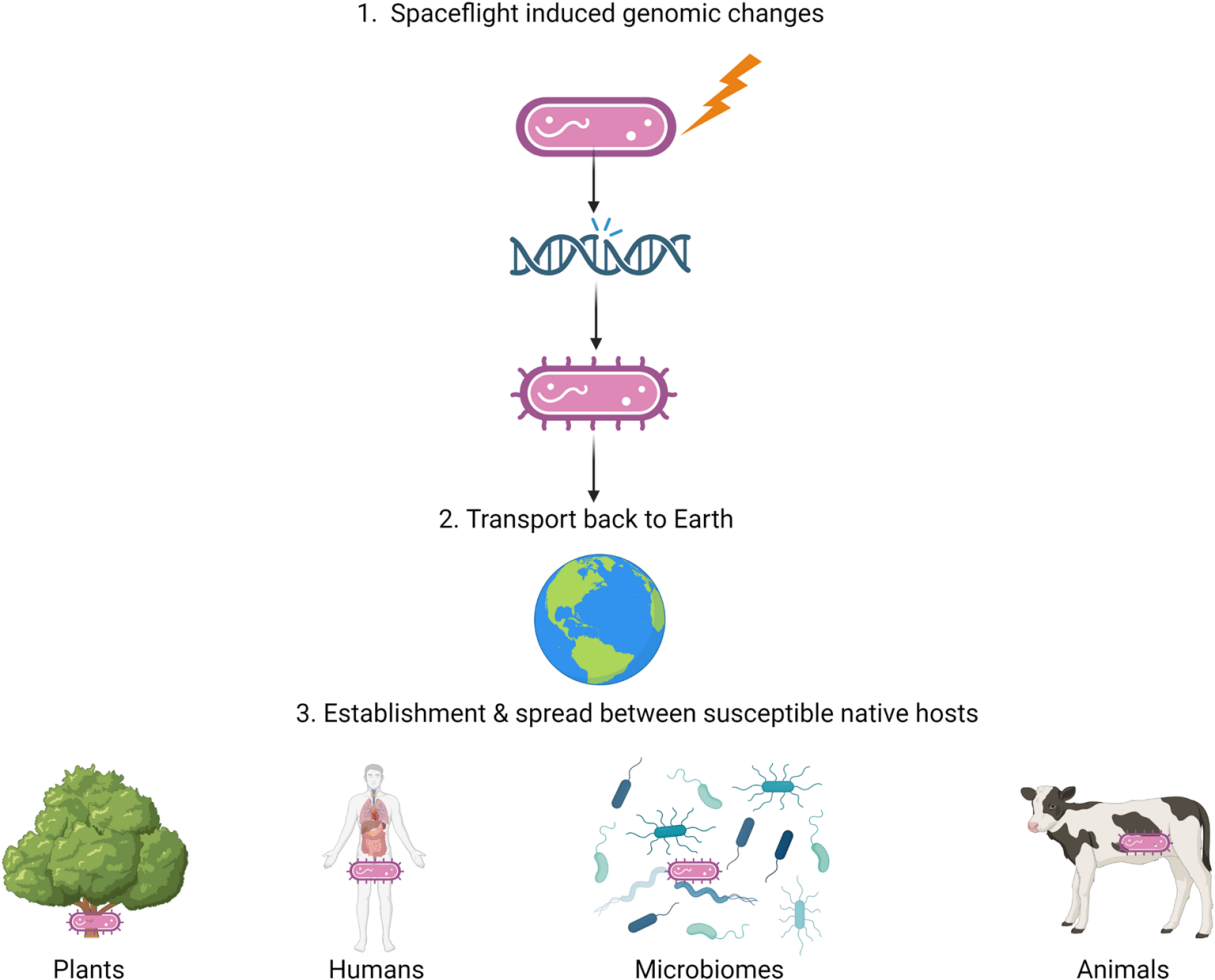
Illustration of a theoretical spaceflight altered invasive species introduction back to Earth.

It is evident that specific bacterial and fungal species adapt and change as an effect of spaceflight conditions. So far, our understanding of the risks (if any) of re-introducing these spaceflight adapted microorganisms back to Earth is very limited. Although several new bacterial species and one bacterial genus have been described on ISS,^129,164^ there were very likely originating from the crew’s own microbiome and cargo-associated contamination. Re-introduction of spaceflight changed microorganisms back to Earth could pose the risk (however small) of a microbiological invasion with environmental and public health consequences. Microbial invasion in established ecosystems is a topic not currently very well understood.^165^ As with invasive plants and animals, the effects of invasive microbes to the composition and functioning of ecosystems is significant.^165^ It has been suggested that invasion consists of four main stages including (i) the transport of invasive species outside of their native environment, (ii) the introduction of invasive species into the new environment, (iii) the establishment of the invasive species, and (iv) the spread of invasive species and subsequent impact on the ecosystem^165^ (Fig. 3). These invasive microorganisms could be pathogenic or non-pathogenic and could be introduced accidentally or purposely. There are several specific examples of such microbial invasions. One includes the invasion of the fungus *Fusarium circinatum,* a key pathogen of *Pinus* species.^MN28^

^**MN28**^A genus of 115 species of evergreen conifers, abundant worldwide.

This plant pathogen was disseminated globally by insect vectors and the wind causing severe pine infections that altered the pine forest composition to oak-dominated woodlands.^166^ Another example includes a north America native racoon species (*Procyon lotor*) which introduced to Europe the nematode *Baylisascaris procyonis,* a parasite that in rare events can cause severe neurologic and ocular disease in humans.^167^ Overall, in the last centuries over 16,000 invasive species introductions have been recorded globally.^168^ In terms of association of invasive microbial species with human health; invasive mirobial specis can either act as a source of new pathogens or amplify other local pathogens.^169^ Microbial species discussed in this review showing high adaptability in spaceflight conditions would be more likely to act as invasive species upon their putative re-introduction to the terrestrial environment. Their putative re-introduction could either occur via crew colonisation during spaceflight or via cargo contamination prior to returning to Earth. A co-ordinated research effort is required to survey strains from microbial species with increased spaceflight adaptability and mitigate the risk (however small) of a spaceflight associated microbial invasive species introduction event.

## Celestial Body Habitation

In addition to spaceflight, a different set of considerations relates to the permanent presence of humans on the surface of other celestial bodies. Within the next few decades this is expected to be true for Mars and the Moon. Proposals for human presence on other planets including Venus, or even Jovian moons (e.g., Callisto) are in place, though such human presence is unlikely within the twenty-first century timeframe.^170^ Possible signs of microbial life have also recently been reported on one of Saturn’s moon Enceladus, in terms of geochemical processes alone being unable to explain the levels of methane measured by the Cassini spacecraft.^171^ The environmental conditions on each of these planets and moons is unique and, therefore, different selective pressures would exist on each one. For the purposes of this review, we will focus on Mars as the most relevant for a permanent human presence in the immediate future. The environmental conditions of the Moon have always been hostile to life, and it is accepted that the Moon would be a highly unlikely place for native microbial life. A review on lunar astrobiology has been previously conducted.^172,173^

On Earth, several Mars Simulation Chambers (MSC)^MN29^

^**MN29**^Temperature and atmosphere regulated chambers replicating conditions met on Mars’s surface.

have been developed to simulate the environmental conditions that will be encountered by the first Mars settlements.^174^ MSCs are stainless steel low-pressure cylindrical chambers which simulate the following five environmental components of Mars’s surface: (i) pressure, (ii) UV radiation, (iii) dust loading, (iv) temperature, and (v) atmospheric gas mixture.^175^ One study used an MSC approach to evaluate the survival on Mars of *E. coli* as a key potential spacecraft contaminant. The main findings related to the ability of *E. coli* cells to survive on Mars soil conditions over a period of 7 days; however, they were not able to replicate. Desiccation (0.71 kPa) was identified as the key limiting factor for the resurrection of viable cells on Mars simulated soil.^176^ The effect on the mycobiome due to human presence in Moon/Mars analogue habitats over a 30-day period has also been investigated.^177^ Human presence appeared to significantly impact fungal diversity with the fungal genera of *Epicoccum*, *Alternaria*, *Pleosporales*,  *Davidiella* and *Cryptococcus*, presenting an increased abundance. These fungal genera are established plant pathogens and human opportunistic pathogens; hence additional studies are required to evaluate their health impact to plants and human is such sealed habitats.^178–181^ A thorough analysis on filamentous fungi from the ISS and from Chernobyl exposed to simulated Mars conditions (SMCs) has also been conducted.^26^ As with previous studies conducted on ISS fungal strains, adaptation to SMCs appeared to occur via alterations of SM production, such as starvation response enzymes. Overall, adaptation to SMCs, as with spaceflight conditions, appears to be species specific for fungi. Like microgravity simulations on Earth, it is not currently possible to exactly replicate the stressors encountered on Mars. Hence, the exact impact of the Martian environmental stressors could be better verified with on-site autonomous or human experiments.

Native microbial life on Mars, although not yet identified is perceived as possible given the Earth like conditions in Mars 3.8 billion years ago.^182^ Robotic exploration to date has purposely avoided the exploration of potential “Special Regions”,^MN30^

**MN30**A region on Mars’s surface with an increased chance of allowing terrestrial organisms to replicate and have the potential to sustain native Martian life.

places on the planet with high chance of being inhabited by extant Martian life.^23^ The main reason relates to forward Planetary Protection policies, aimed at protecting Mars with microbial contamination originating from Earth^138^. Given the radiation dosage reaching the surface of Mars, microbial life if it has survived, is likely to be buried below the surface. Simulating ionising radiation conditions encountered on Mars, a recent study estimated that the extremophilic *Deinococcus radiodurans* could survive over 100 million years buried 10 m below Mars’s surface.^183^ This finding was quantified using cell manganese antioxidant accumulation as a sign of tolerance to different forms of radiation. This raises the possibility that if microbial life ever existed on Mars, it could still be possibly viable with unchartered potential effects and interactions with Earth’s biosphere. There is the possibility, therefore, however small, that the first Mars explorers could be exposed to Mars native microbial life. To mitigate any risk of accidental exposure, facilities similar to Biosafety Level 4 (BSL-4) laboratories used for studying highly pathogenic viruses on Earth, should be pre-planned and become immediately accessible before any microbial identification experiments take place by human personnel. An understanding of how pathogens adapt and behave in Martian environmental conditions is also required prior to human settlement to mitigate any public health risks to the first human settlements.

## Conclusion

The future path of humanity appears to progressively expand beyond the terrestrial environment. Managing the microbiological and infectious risks and associated fears arising from this are key to safety, sustainability, and public and overall government support for this endeavour. As outlined, we currently have very limited understanding of many of the risks in relation to the microbiome, the factors that drive its adaptation and of the host factors that contribute to susceptibility to colonisation and infection. In relation to causality for changes in microorganisms and hosts associated with space travel, attention has naturally focused on microgravity and radiation exposure but as outlined throughout this review there are many other factors that may be as important or more important in shaping the microbiome of the space vehicle and the response of the host to that environment. Review and reflection on the current literature in the framework of the risks and variables points to several conclusions.

There are extensive gaps in our understanding of how space travel reshapes terrestrial microorganisms and impacts on host resistance to infection. Human space travel introduces terrestrial organisms into space environments and organisms from space environments back into terrestrial environments. This exchange will increase along with an increased human presence in space. In that context, surveillance of space environments and of infection or other microbial impacts associated with space travel is important. The risk of introducing a harmful microorganism that has adapted within a spacecraft environment and is capable of dissemination in the terrestrial environment is very low but requires caution and surveillance regardless.

The interior of a spacecraft will inevitably develop a microbiome that will adapt over time to that environment. Protocols that attempt to maintain low-biomass of the spacecraft environment are likely to drive that adaptation towards a unique closed habitat that is tolerant of the disinfectants and the antimicrobial coating of surfaces used to maintain a clean room type environment. Processes more like those that apply in a clean domestic setting may lead to a more terrestrial like microbiome mitigating the effects of ultra-clean environments to human health. A balance between a more ‘natural’ spaceflight environment and planetary protection from human contamination can be managed and achieved.

Human travel to other celestial bodies will inevitably lead to introduction of microorganisms to those environments. Local containment will likely be very challenging if the environment can support growth of terrestrial microbes. How terrestrial microorganisms will adapt and change in these extra-terrestrial environments is not currently understood. Furthermore, there is the risk, however small, that human settlers might encounter extra-terrestrial microbial life. Celestial body habitation requires thorough planning, containment and mitigation strategies from a medical astro-microbiology perspective. Overall, a framework carefully built around the microbiological and infectious disease considerations would ensure the long-term viability of our multi-planetary aspirations.
